# School climate and adolescents’ prosocial behavior: the mediating role of perceived social support and resilience

**DOI:** 10.3389/fpsyg.2023.1095566

**Published:** 2023-07-06

**Authors:** Yun Luo, Tangsheng Ma, Yuting Deng

**Affiliations:** ^1^School of Education, Zhaoqing University, Zhaoqing, China; ^2^Department of Psychology and Research Center of Adolescennt Psychology and Behavior, School of Education, Guangzhou University, Guangzhou, China

**Keywords:** school climate, prosocial behavior, perceive social support, psychological resilience, adolescent

## Abstract

Prosocial behavior is crucial for adolescent socialization and plays a positive role in all aspects of adolescent development. Based on ecosystem theory and self-determination theory, this study aimed to explore the relationship among school climate, perceived social support, psychological resilience, and prosocial behavior. With 1,688 high school students being sampled, we utilized the Perceived School Climate Questionnaire, the Perceived Social Support Scale, the Chinese Version of Mental Resilience Scale, and the Adolescent Prosocial Tendency Scale. The results showed that: (1) school climate, perceived social support, psychological resilience, and prosocial behavior were all positively correlated; (2) perceived social support and psychological resilience could independently mediate the relationship between school climate and prosocial behavior; these two mediating variables could develop a chain mediation effect to influence the link between school climate and prosocial behavior.

## 1. Introduction

Prosocial behaviors, such as sharing, helping, and caring, are defined as voluntary actions and beneficial to others and society (Eisenberg et al., 2006). This type of behavior is key in adolescents’ socialization endeavors and plays a positive role in all aspects of their development; specifically, prosocial behavior can promote academic performance, self-esteem, sense of happiness, and social adaptation in adolescents (Renouf et al., 2010; Aknin et al., 2012; Zuffianò et al., 2014; Caprara et al., 2015). The study found that participation in prosocial behavior can reduce the negative impact of daily stress on mental health and emotion (Raposa et al., 2016). The cultivation of young students’ prosocial behavior is not only related to the formation of social responsibility and moral behavior, but also related to the development, progress, harmony and stability of society (Guo, 2017). We also know that adolescence is a relatively rapid period during which we develop ourselves both socially and morally (Karmakar, 2017), making it so that exploring the underlying mechanisms influencing adolescents’ prosocial behavior becomes a matter of high practical significance.

Ecosystem theory (ET) points out that human development is influenced not only by own biological and personality characteristics but also by the environment (Bronfenbrenner, 1977; Bronfenbrenner and Morris, 2006). The study found that fostering prosocial behavior in school helps students learn and adapt well, while protecting them from the negative consequences of aggression, including peer rejection and anti-social behavior (Caprara et al., 2015). In concordance with this positing, many researchers have recently come to emphasize the influence of school environment on adolescents’ positive development (e.g., the cultivation of prosociality), with school climate being a major concept in this context (Kanacri et al., 2017). As a unique attribute of related organizations, school climate refers to the psychosocial atmosphere of the school as perceived by its members (Cohen et al., 2009; Jia et al., 2009); this concept can be broken down into the three dimensions of interpersonal, teaching, and organizational atmospheres (Way et al., 2007; Danielsen et al., 2010; Wang and Dishion, 2012).

First, regarding interpersonal atmosphere, studies like those by Zimmer-Gembeck et al. (2005) and Barry and Wentzel (2006) showed that positive peer relationship can effectively predict prosocial behavior. According to previous research reports, children tend to imitate their peers after observing their peers’ prosocial behavior; In other words, the prosocial peer model is likely to trigger children’s prosocial behavior (Kato-Shimizu et al., 2013). They also demonstrated that early peer acceptance and positive peer groups are closely related to late adolescents being enthusiastic about engaging in prosocial behaviors. Concomitantly, in student-teacher relationships, scholars have shown that the following can promote prosocial behavior: teachers’ expectation and evaluation (Eisenberg et al., 2006), and the attachment and support between teachers and students (Chang et al., 2004). The close relationship between teachers and students plays an important role in internalizing pro social values (Kuswendi, 2019).

Second, regarding teaching atmosphere, research shows that, through direct teaching and classroom-based education, teachers promote student learning and the internalization of some paradigms related to prosocial behavior—a context conducive to this type of behavior. Positive and caring teaching practice and classroom atmosphere will promote students’ prosocial behavior (Barr et al., 2007). For example, after 3 years of intervention research, Mooij (2011) found that students who experienced prosocial classroom teaching (experimental group) showed more prosocial behaviors than the non-experimental group.

Third, regarding organizational atmosphere, Schwartz (1975) emphasized the role of social norm consciousness (at the individual level) in altruistic behavior; specifically, although school discipline and rules (i.e., two social norms) both have a restraining effect on student behavior, both school spirit and class spirit have a subtle conducive effect. Further, research has shown that the moral atmosphere of the school is significantly related to prosocial behavior (Spruit et al., 2019). Namely, deeply exploring the relationship between the moral atmosphere of the school and prosocial behaviors may be a key action to better understand how to facilitate student engagement in prosocial behaviors. In summary, the literature shows that different elements of school climate affect individual prosocial behavior in different ways.

### 1.1. The mediating role of resilience

According to ET, both school climate (i.e., external environment) and adolescents’ personal characteristics influence their prosocial behaviors. Numerous studies have demonstrated that students’ psychological characteristics mediate the link between school factors and positive development results (Wang, 2009; Rutten et al., 2012; Jeong and Bae, 2020; Li and Qin, 2020). Among psychological factors, resilience has been receiving much research attention, deemed as an important psychological factor (Xiaonan and Jianxin, 2007), and may be an influencing factor of the effect of school climate on prosocial behavior. In addition, both the positive adolescent development view theory and the development self-system theory describe that the mechanism through which the environment influences development outcomes may be modified by individual behavior and self-system (Tian et al., 2015). Social cognitive theory believes that environment, individual cognitive factors and behavior interact, that is, environmental factors (such as school atmosphere) will affect individual cognitive factors (such as psychological resilience), and individual cognitive factors (such as psychological resilience) can affect individual behavior (prosocial behavior). From this, it can be concluded that environmental factors → individual cognitive factors → individual behavior research path. Therefore, resilience may be a mediator in the association between school environment and prosocial behavior.

For one, a positive school climate can improve individual resilience. It has been described that positive school environment (e.g., positive teacher-student relationships, peer relationships, and high school life satisfaction) is a protective factor of resilience, being able to improve individual resilience (Masten and Coatsworth, 1998; Luthar et al., 2000). Positive peer relationships, social support, and supportive environments are considered external characteristics that can increase resilience (Bean, 2019). In an international cross-sectional study on the resilience of migrant and non-migrant youth, it was pointed out that the factors related to the resilience of children and adolescents include personal characteristics such as active caregivers, family and peer relationships, religion, school climate, and self-regulation and coping skills (Gatt et al., 2020). Indeed, in a longitudinal study on school climate and adolescents’ social–emotional health, Wong et al. (2021) showed that a positive school environment, interpersonal relationships, and campus discipline all helped improve adolescents’ perseverance and self-efficacy.

For another, adolescents with higher resilience are more likely to perform prosocial behaviors. High resilience means that individuals possess more resources, and some scholars have pointed out that the more resources one has, the more benefits one experiences in own development. For example, a study showed that when individuals high in resilience are faced with complex situations in which they can be of assistance, they are able to use high levels of energy and resources in order to help others (Benson et al., 2006). Other scholars have found that resilience, as an important positive psychological trait of individual development, can help individuals maintain high empathy; empathy and resilience can, in turn, reliably predict altruism, denoting that those with high resilience are more likely to show altruistic behaviors (Werner, 1992; Leontopoulou, 2010).

In addition, positive emotions were manifested to mediate the link between school environment and development outcomes (Lee et al., 2021), with optimism (i.e., a major factor of resilience) significantly influencing this mediation effect. Yanhui et al. (2017) further found that highly optimistic individuals pay more attention to others’ status, needs, and are more willing to help others through actions/behaviors. Further, the school climate characterized by caring relationships, meaningful participation, collaboration, high expectations, and shared norms has been identified as contributing to educational resilience. Therefore, those students who live in a safe and supportive environment and can experience understanding, care, tolerance and support also have strong motivation to participate in these behaviors when they see other team members participating in the practice of solidarity, cooperation and prosocial behaviors (Cefai, 2007). Therefore, we figured that resilience would mediate the influence of school climate on adolescents’ prosocial behavior.

### 1.2. The mediating role of perceived social support

Although ET expects adolescent development to be influenced by school climate, it does not specify the underlying mechanisms of this influence. Self-determination theory (SDT) emphasizes that we need to establish positive relationships with others, and that the satisfaction of this relationship-related need is conducive to constructive individual development (Deci and Ryan, 2000). In view of the literature, we hypothesized that, in the school setting, adolescents may have their relationship-related needs be well met upon experiencing care and encouragement from teachers and mutual care and comfort from peers, which may thereby generate positive emotions and cognition, as well as create the perception of having positive interpersonal relationships and social support (Inguglia et al., 2019; Rodrigues et al., 2020; Kaefer and Chiviacowsky, 2021). Now, because external influences usually need to pass through own cognition and interpretation to be able to work, we believe that perceptions about own interpersonal relationships may have a helpful effect on behaviors (Virtue et al., 2014); namely, individuals’ perceived social support may play an indispensable role in the engagement in prosocial behaviors, perhaps even one greater role than that of the environment. Two studies depicted this mechanism, demonstrating that perceived social support had a greater function on individuals’ behaviors than the environment (Vaux, 1990; Rodríguez-Fernández et al., 2021). For instance, talking to friends about one’s troubles and emotions was far more supportive than just having a friend without communication. When individuals perceive high levels of social support, they may pay more attention to the needs of others, thus promoting helping behaviors (Li et al., 2021). Therefore, perceived social support may be an important mediator in the effect of school environment on prosocial behavior.

First, in the school environment, adolescents’ social relations mainly concentrate on teachers and peers, from which they can receive emotional, informational, and self-esteem support, among others (Rad et al., 2014). Teachers’ support, parents’ encouragement and peers’ support are protective factors for children and adolescents’ prosocial behavior (Lee et al., 2014). Further, compared with adolescents with conflictual teacher-student relationships, those with intimate teacher-student relationships showed better mental health indices (e.g., higher subjective well-being and self-esteem; Huang et al., 2018; Farhah et al., 2021). When individuals perceive a good and intimate interpersonal environment and close organizational relationships, they develop a strong sense of belonging, feeling that people are trustworthy and that the world is warm and beautiful, so they will treat others gently, which promotes altruistic behavior (De Guzman et al., 2012).

Second, individuals with high perceived social support may be more likely to show prosocial behavior. On the one hand, individuals’ positive feelings have been able to promote own prosocial (helping) behavior (Telle and Pfister, 2015). On the other hand, perceived social support may be an index of own perception of prosocial behaviors, implying that when the first is high, the second may be more likely to happen (Maltseva, 2012). Meanwhile, the lack of perceived social support and the experience of social exclusion have been associated with a decreased engagement in prosocial behaviors (Twenge et al., 2007). Behaviorism theory mentions that individual behavior is formed and developed through the interaction between the individual and the environment. As a very important external environmental resource available to individuals, social support will not only affect health level, but also affect behavior patterns, which includes the impact on prosocial behavior. Research shows that when individuals face the COVID-19, social support plays a buffer role, thus protecting the mental health of adolescents. When they feel stable about their support and access to resources, they are more inclined to participate in prosocial behavior to repay others and society (Xue et al., 2022).

Third, empirical research on adolescents’ positive development showed that perceived social support mediates the link between positive parent–child relationships (i.e., development environment) and higher levels of social adaptation (i.e., positive development outcomes) (Anan and Barnett, 1999; Pace et al., 2016). Thus, we conjecture that perceived social support, as an essential perceptual process of individuals (Rodríguez-Fernández et al., 2021), mediates the relationship between school climate and prosocial behaviors.

### 1.3. The present study

We believe that an examination integrating the two aforementioned mediation mechanisms of the link between school climate and prosocial behaviors has scientific and practical significance. Multiple mediation models are more comprehensive than their simple counterparts because they enable for: calculating and comparing the effect sizes of different models; reducing errors to a certain extent (Preacher and Hayes, 2008); integrating theories and considering the research question from multiple perspectives (MacKinnon et al., 2007).

Multiple mediation models can take on two forms: parallel or chain mediation (Hayes, 2009). A meta-analysis of studies on social support and resilience showed that these two variables are closely related (Xue et al., 2014), leading us to exclude considerations about parallel mediation between resilience and perceived social support. This left us with a chain mediation model, wherein one variable needs to come before the other in the influence chain. According to the following two arguments, we hypothesized that perceived social support would precede resilience (i.e., perceived social support → resilience). First, perceived social support is a perceptual process and resilience is a psychological characteristic, and researchers show that perceptual processes tend to work faster than formation processes for a personal characteristic (Masten and Coatsworth, 1998). Second, positive social relationships have been described as important protective factors of resilience (Luthar et al., 2000), and the chain mediation model we propose conforms with social support theory and its main effect model, which posits that positive social relations are beneficial to the healthy development of individuals (Rad et al., 2014). Therefore, we aimed to test the validity of a chain mediation model with perceived social support preceding resilience.

**Figure 1:**
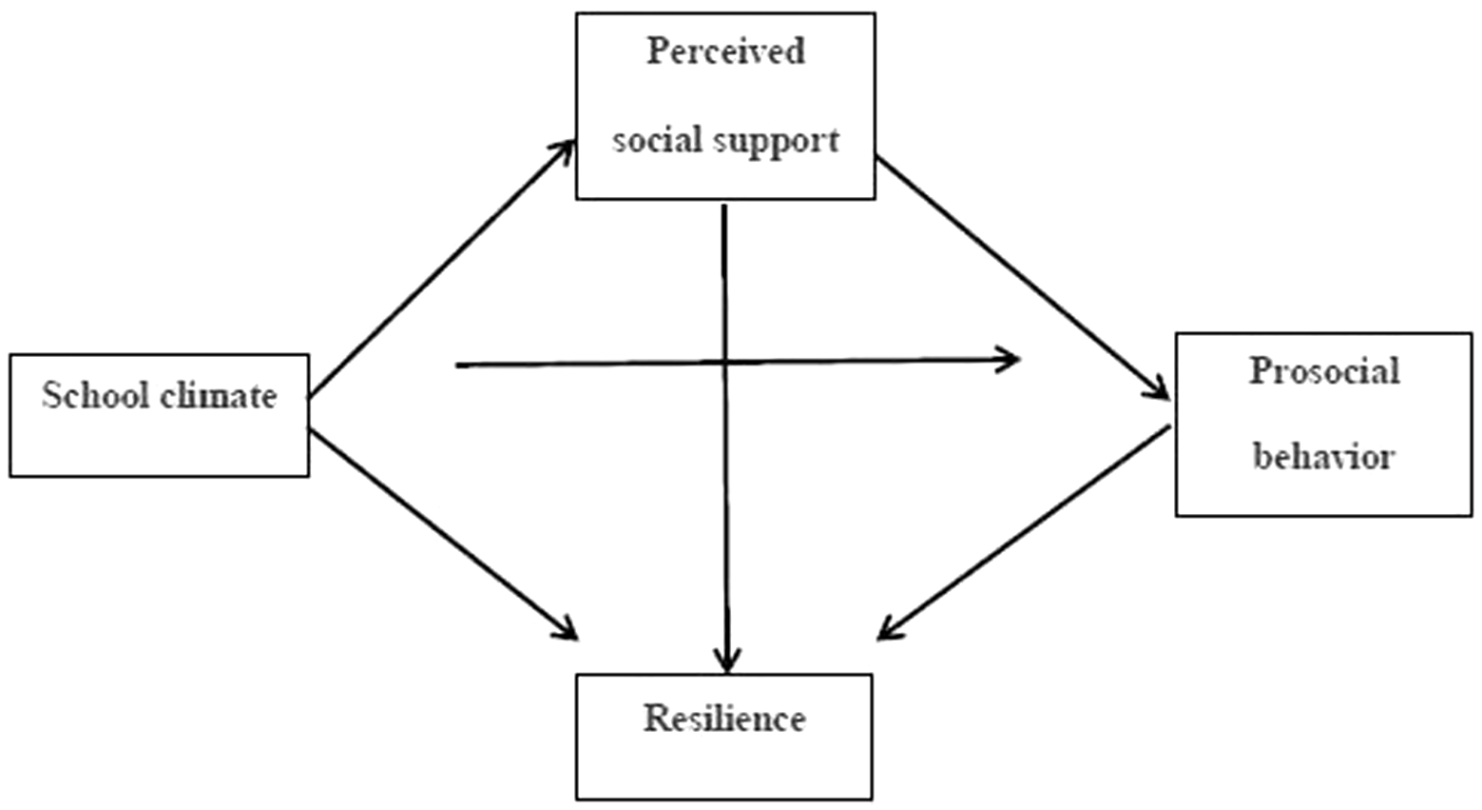
Hypothesized research model.

In summary, in accordance with ET and SDT, this study aimed to discuss the link between school climate and adolescents’ prosocial behavior, and the mediation effects of perceived social support and resilience. Accordingly, we put forward the following hypotheses: positive school climate has a prominent positive predictive effect on adolescents’ prosocial behaviors (Hypothesis 1, H1); perceived social support mediates the association between school climate and adolescents’ prosocial behavior (Hypothesis 2, H2); resilience mediates the association between school climate and adolescents’ prosocial behavior (Hypothesis 3, H3); perceived social support precedes resilience in the chain mediation model (Hypothesis 4, H4). Our study model is presented in Figure 1.

## 2. Methods

### 2.1. Participants

In this study, Students from grade-one and grade-two students from different high schools in Dongguan city, China, Data were collected through “*wenjuanxing,”* which is a Chinese platform with functions similar to those of Amazon M Turk. Before administering the questionnaires, informed consent was obtained from the students. Students were invited to participate voluntarily and complete the questionnaires anonymously, and after deleting 15 invalid questionnaires according to lie detection questions and unfinished questionnaires, 1,688 valid questionnaires were collected. In our final sample, the average age was 14–19 (*M_age_* = 16.76 years, *SD* = 0.85, 745 boys and 943 girls) and there were 792 grade-one and 896 grade-two students. Regarding their fathers’ and mothers’ education level, a large proportion of fathers (54%, *n* = 911) and mothers (48.9%, *n* = 825) had junior high school education, some had senior high school education (fathers: 23.2%, *n* = 392; mothers: 18.1%, *n* = 305), and a few had at least higher education (fathers: 5.2%, *n* = 89; mothers: 2.9%, *n* = 49).

### 2.2. Measures

#### 2.2.1. School climate

To assess school climate, we used the 38-item School Climate Questionnaire (PSCI-M) developed by Minggui and Yibing (2006). It comprises five subscales: teacher-student relationship (9 items), classmate relationship (7 items), academic pressure (8 items, which are reverse scored), order and discipline (7 items), and development diversity (7 items). A sample item is ‘Schools encourage students to participate in activities of their interest.’ It is responded on a four-point scale that ranges from 1–4 (completely inconsistent–completely consistent), and the higher the total score (the sum of all dimensions is the total score), the more positive the perception of school climate. In this study, the internal consistency coefficient of the total scale was 0.90.

#### 2.2.2. Perceived social support

To assess perceived social support, we used the Perceived Social Support Scale (PSSS) with 12 items, which was developed by Blumenthal et al. (1987). It comprises three subscales: family support (3 items), friend support (5 items), and other support (4 items). Sample items include: ‘I can get emotional help and support from my family when I need it.’ It is responded on a seven-point scale that ranges from 1–7 (extremely disagreeing–extremely agreeing), where the higher the total score (the sum of all dimensions is the total score), the higher the social support perceived by the individual. In this study, the internal consistency coefficients for the total scale and each subscale (i.e., family support, friend support, and other support) were 0.95, 0.90, 0.89, and 0.91, respectively.

#### 2.2.3. Resilience

To assess resilience, we used the 25-item Chinese version of the Mental Resilience Scale (CD-RISC) revised by Xiaonan and Jianxin (2007). It comprises three subscales: tenacity (13 items), self-improvement (8 items), and optimism (4 items). An example item is ‘I work hard to reach the goal.’ It is responded on a five-point scale that ranges from 1–5 points (very inconsistent–very consistent), where the higher the score (the sum of all dimensions is the total score), the higher the resilience. In this study, the internal consistency coefficients for the total scale and each subscale (i.e., tenacity, self-improvement, and optimism) were 0.96, 0.93, 0.87, and 0.83, respectively.

#### 2.2.4. Prosocial behavior

To assess prosocial behavior, we used the 26-item Adolescent Prosocial Tendency Scale (PTM) revised by Yu et al. (2007). It comprises six subscales: emotion (5 items), compliance (5 items), altruism (4 items), anonymity (5 items), openness (4 items), and urgency (3 items). An example item is ‘When I am asked for help, I rarely refuse.’ It is responded on a five-point scale that ranges from 1–5 points (completely inconsistent–completely consistent), where the higher the score (the sum of all dimensions is the total score), the higher the prosocial behavior tendency. In this study, the internal consistency coefficients for the total scale and each subscale (i.e., emotion, compliance, altruism, anonymity, openness, and urgency) were 0.96, 0.84, 0.85, 0.85, 0.88, 0.83, and 0.79, respectively.

### 2.3. Procedures

First, after obtaining the informed consent of the school leaders and students, a meeting was held with the school leaders and teachers to explain the research objectives and procedures in detail. Second, the researchers went to the classes that agreed to participate, and conducted a questionnaire survey among the students who submitted the informed consent signed by their parents or legal guardians. The questionnaire was completed collectively in the participants’ own classroom during class time (30 min at most). Third, survey application was managed by members of the research team, who solved any questions that students had in the process of filling out the questionnaires. In order to avoid the deviation of social expectations, students were not informed of research purposes, their answers were guaranteed to be anonymous, and their participation was completely voluntary.

### 2.4. Data analysis

First, the raw data was entered in the SPSS sheet and primary analysis, then eliminate invalid questionnaires according to lie detection questions, and purify missing values and outlier to finally obtain valid questionnaires. Second, the common method deviation test was carried out to ensure that there is no serious collinearity problem between variables. The specific operation is to use SPSS to conduct Harman single factor common method deviation test, and extract all items of school climate, perceived social support, resilience and prosocial behavior into exploratory factor analysis. The results indicate that there are a total of 12 factors with eigenvalues greater than 1, and the first factor explains a total variance of 28.97%, which is less than the critical value of 40%, indicating that there is no serious common method bias in this study. Third, two structural equation models were constructed by AMOS 21 software, and the resulting model with better fitting degree was retained.

## 3. Results

### 3.1. Preliminary analyses

As seen from Table 1, school climate, perceived social support, resilience, and prosocial behavior were positively associated with each other. Table 1 provides the means, standard deviations, and correlations between the research variables.

**Table 1:** Means, standard deviations, and correlation coefficients of each variable.

	1	2	3	4
1. School climate	—			
2. Perceived social support	0.474^***^	—		
3. Resilience	0.315^***^	0.568^***^	—	
4. Prosocial behavior	0.327^***^	0.581^***^	0.640^***^	—
*M*	2.688	4.755	3.322	3.508
*SD*	0.342	1.02	0.605	0.573

^***^Means *p* < 0.001, the same below.

### 3.2. Structural modeling

In this study, the latent variables were school climate, perceived social support, resilience, and prosocial behavior. For school climate, the five subscales of the PSCI-M were deemed as observable variables; for resilience, they were the three subscales of the CD-RISC; for prosocial behavior, they were the six subscales of the PTM. For perceived social support, only two subscales of the PSSS (i.e., friend support, and other support) were deemed as observable variables; this is because we explored interpersonal relationships originating in school environments, wherein family support tends to be non-abundant.

In conformity with our hypotheses, we established two mediation models and determined the optimal one by analyzing model fit indices. In Model 1 (partial chain mediation model), we used school climate as the independent variable, prosocial behavior as the dependent variable, and perceived social support and resilience as the mediating variables; it showed that school climate points to prosocial behavior, perceived social support points to resilience, and that both perceived social support and resilience point to prosocial behavior. In Model 2, we depicted the complete chain mediation model. In conformity with Model 1, the path coefficient from school climate to prosocial behavior was limited to 0.

Hu and Bentler (1998) and McDonald and Ho (2002) propose that a model has good fit to the data when the CFI and TLI are >0.90 and the SRMR and RMSEA are <0.08. Hence, Model 1 and Model 2 showed a good fit to the data (Table 2). Then, we compared the nested models of the two models, with results showing that the χ ^2^ increased significantly in Model 1, *χ* ^2^ (1,688) = 9.23, *p <* 0.001. That is, Model 1 showed a superior fit to the data compared to Model 2.

**Table 2:** Model fit indices of the influence of school climate on prosocial behavior.

Model	χ^2^	*df*	RMSEA	SRMR	TLI	CFI
Model 1	705.84	154	0.046	0.044	0.971	0.976
Model 2	715.07	155	0.046	0.044	0.971	0.976

### 3.3. Mediation effect test

**Figure 2:**
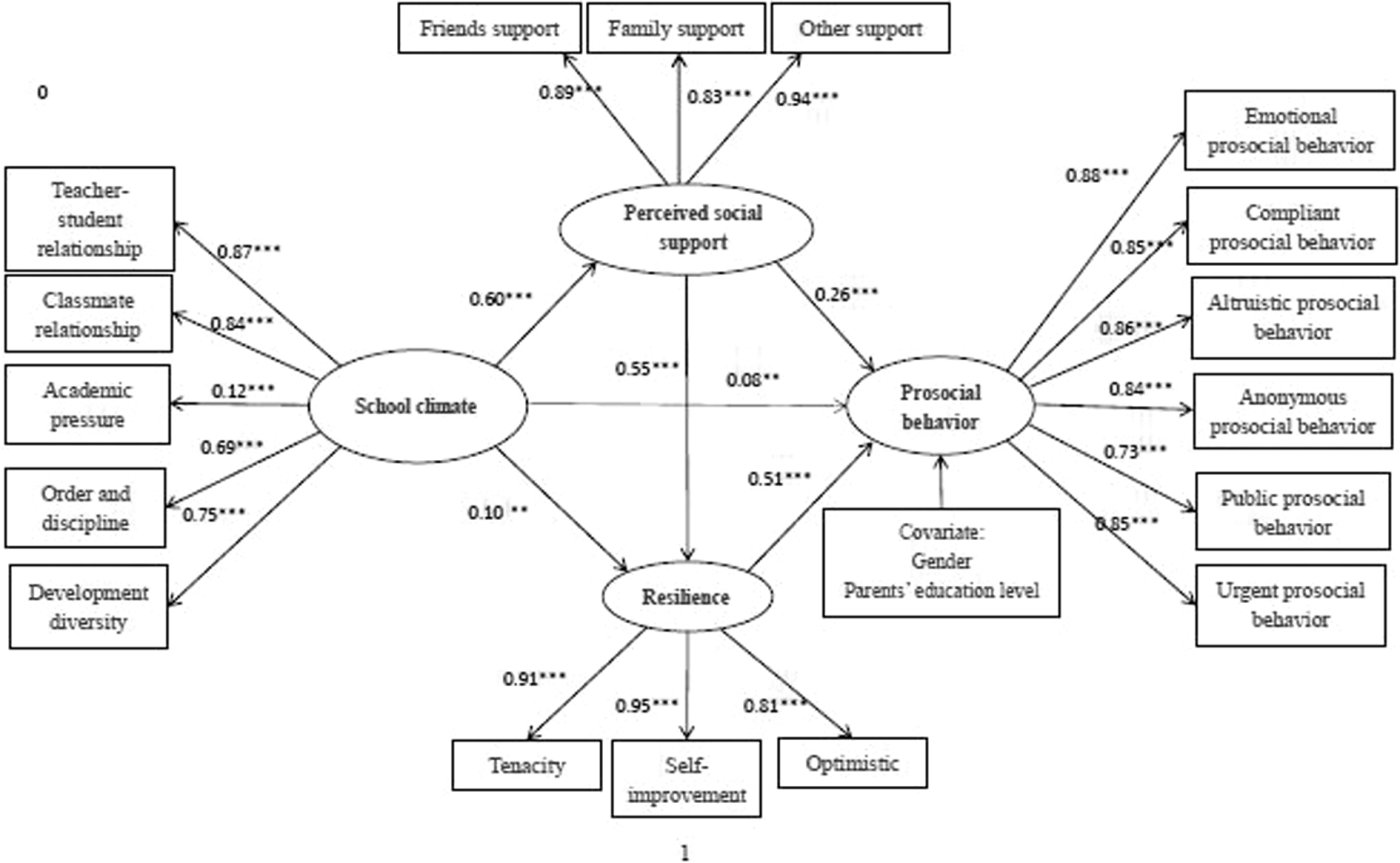
Chain mediation model diagram.

According to Model 1, the path diagram of the influence of school climate, perceived social support, resilience on prosocial behavior (Figure 2), school climate positively predicts prosocial behavior (*β* = 0.08, *p* < 0.01), supporting H1.

School climate positively predicts perceived social support (*β* = 0.60, *p* < 0.001), and perceived social support positively predicts prosocial behavior (*β* = 0.26, *p* < 0.001), supporting H2 and indicating that school climate can indirectly influence prosocial behavior through perceived social support.

School climate positively predicts resilience (*β* = 0.10, *p* < 0.01), and resilience positively predicts prosocial behavior (*β* = 0.51, *p* < 0.001), showing that school climate can indirectly influence prosocial behavior through resilience, supporting H3.

Perceived social support positively predicts resilience (*β* = 0.55, *p* < 0.001), indicating that school climate can indirectly affect prosocial behavior through the effect of perceived social support and resilience, which supports H4.

Grounded in prior research (Yuan and MacKinnon, 2009; Hayes and Scharkow, 2013), to test the mediating effects in our hypotheses and research model, we used the bias-corrected nonparametric percentile Bootstrap method. Specifically, through repeated random sampling, we employed 5,000 Bootstrap samples from the original data (*N* = 1,688), generating an approximate sampling distribution. We estimated 95% confidence intervals (CIs) of the mediating effects by percentile. The results are shown in Figure 2 and Table 3.

**Table 3:** Mediating effect analysis.

Content	Path relationship	Effect size	95%CI
Indirect path 1	School climate →Perceived social support →Prosocial behavior	0.16	[0.010, 0.21]
Indirect path 2	School climate →Resilience →Prosocial behavior	0.17	[0.11, 0.23]
Chain mediation	School climate →Perceived social support →Resilience →Prosocial behavior	0.05	[0.01, 0.10]
Direct effect	School climate → Prosocial behavior	0.08	[0.04, 0.12]
Total effect		0.46	

In indirect effect path 1 (school climate → perceived social support → prosocial behavior), the 95% CI was [0.10, 0.21]; since it did not include a 0, we deemed the path effect as significant, and the ratio of the indirect to the total effect was 34.78%. Further, in indirect effect path 2 (school climate → resilience → prosocial behavior), the 95% CI was [0.01, 0.10]; since it did not include a 0, we deemed the path effect as significant, and the ratio of the indirect to the total effect was 10.87%. In the chain mediation path (school climate → perceived social support → resilience → prosocial behavior), the 95% CI was [0.11, 0.23]; since it did not include a 0, we deemed the path effect as significant, and the ratio of the indirect to the total effect was 36.96%.

## 4. Discussion

Our findings demonstrate the significant positive correlation between school climate and prosocial behavior, indicating that the better the school climate, the higher the tendency of students to engage in prosocial behaviors. This supported H1 and is consistent with prior evidence on the topic (Kanacri et al., 2017; Manzano-Sánchez et al., 2021). To explain this result, first, researchers have shown that positive relationships represent reciprocal and positive social behaviors, which usually comprise engagement in prosocial behaviors (Eberly and Montemayor, 1998). Therefore, positive social support (e.g., positive teacher-student and peer relationships) can provide a positive environment for prosocial behaviors to thrive (Obsuth et al., 2016). This provides insights on the significance of the effect of school-related interpersonal factors on adolescents’ development.

Second, a positive and democratic school environment can promote students’ prosocial behavior (Lai et al., 2015). In a positive school environment, the norms will revolve around and advocate prosocial behaviors, promoting cooperation, spontaneous engagement in prosocial behavior, and supportive ties among students (Battistich et al., 2004). School supervision and disciplinary punishment have also been shown to positively influence prosocial behavior in adolescents (Cheung and Ngai, 2015). Therefore, the moral and organizational atmospheres of schools also play an important role in adolescent prosocial behavior.

We also discovered that perceived social support mediated the connection between school climate and adolescents’ prosocial behavior, supporting H2. This may be because, first, adolescents change physically and psychologically—and also face various challenges—after entering and during high school. During high school, the tendency is for adolescents to begin to have specific and novel interpersonal needs (e.g., caring for others and mutual support), individual needs (e.g., self-expression and freedom of choice), and ability-related needs (e.g., self-growth skills), among other needs related to other topics (Roeser et al., 2000; Eccles and Roeser, 2011). In light of prior research, we believe that a positive school climate (e.g., prosocial peer communication and positive teacher-student relationships) is conducive to meeting these psychological needs (Inguglia et al., 2019; Rodrigues et al., 2020; Kaefer and Chiviacowsky, 2021). Further, these results support the predictions in stage-environment fit theory, in that the ability of people to reach specific stages of development is closely related to whether the social environment can meet their individual needs (Booth and Gerard, 2014). Second, it may be that individuals with high perceived social support will engage in more prosocial behaviors; indeed, research shows that positive social relationships can facilitate various positive development results, such as self-efficacy, empathy, and positive emotions (Zhu et al., 2013; Liu et al., 2020; Chen and Xu, 2021), which are all facilitators of prosocial behaviors in adolescents.

Our results also demonstrated that resilience mediates the link between school climate and adolescents’ prosocial behavior, supporting H3. This finding supports the development resource theory, which postulates that adolescents’ external (ecological characteristics) and internal resources (personal skills and abilities) impact their positive development (Benson et al., 2006). The theory also posits that these resources are dynamically interconnected, can prevent high-risk behaviors, and improve other related factors in different ways. School climate, represented by variables such as harmonious/loving interpersonal relationships, is a key factor among external resources in development resource theory. Adequate external resources are an important source of internal resources for individual development (Benson et al., 2006). For instance, schools can serve as protective factors by providing opportunities and rewards for social adaptability and personality development, and resilience is an important part of individual ability development (Luthar et al., 2000; Steinhausen and Metzke, 2001). Therefore, a positive school climate can improve student resilience, and this effect has been shown to be relevant in the short-term and have certain stability in the long-term (Moore and Glei, 1995). Meanwhile, with the continuous improvement of resilience, adolescents’ prosocial behavior tendencies may also be improved. For example, Zuffianò et al. (2014) and Atkins and Donnelly (2005) found that resilience is closely related to adolescents’ prosocial traits and voluntary behavior. Therefore, a positive school climate can improve resilience in adolescents, enabling them to perform more prosocial behaviors.

Our results also confirmed the chain mediation of perceived social support and resilience on the relationship between school climate and adolescents’ prosocial behavior, supporting H4. This chain mediation model supports the “scenario-process-result” model in prior research (Roeser et al., 1996); this past model demonstrated that situational factors in schools (e.g., school climate) can influence students’ psychological processes (e.g., perceived social support and resilience), thereby affecting the mediation that leads to developmental outcomes (e.g., prosocial behavior). These results and discussions highlight the need to think highly of the influence of school environment on adolescents.

Further, we may imply that the longer adolescents stay in school, the greater the influence of the school environment; the more students receive support and positive feedback from teachers, the more they will get along with teachers; the more they receive care from peers, the more they will feel secure in their interactions with peers. In sum, these remarks show that a positive school interpersonal climate can make adolescents perceive that they receive more social support (Demaray and Malecki, 2002). On the topic, ET posits that the link between perceived social support (i.e., a psychological construct) and individual development is closer than that between the latter and objective social support (i.e., the actual support people receive) (Bronfenbrenner, 1977). These discussions demonstrate that high perceived social support can facilitate the development of positive and constructive personal characteristics (e.g., resilience), laying the foundation for adolescents’ positive development.

## 5. Conclusion

Overall, from the ecosystem theory and SDT perspectives, the current study makes a significant and novel contribution to adolescent prosocial behavior research by identifying one pathway linking school climate to adolescent prosocial behavior. We believe that our results may help stakeholders in the positive development of adolescents to make more well-informed decisions when developing strategies (related to school environment and climate) to promote prosocial behavior in adolescents.

Despite these strengths, several limitations of this study should be noted. First, it used a cross-sectional design, hindering our ability to investigate the dynamic relationship between variables across time. Therefore, subsequent studies can collect longitudinal data and carry out follow-up research. Second, when selecting samples, our study adopted random cluster sampling, the questionnaire survey was only conducted among students from one school, and the sampling scope can be expanded in future research. Third, the research is conducted under the background of Chinese culture, and the conclusions of this study may not be widely generalizable. Researchers can perform future cross-cultural studies to explore the relationship between school climate and prosocial behavior from a more macroscopic perspective.

## Data availability statement

The raw data supporting the conclusions of this article will be made available by the authors, without undue reservation.

## Author contributions

YL: conceptualization, methodology, investigation, formal analysis, writing—original draft, and writing—reviewing and editing. TM: methodology, formal analysis, and writing—original draft. YD: data curation and writing—reviewing. All authors contributed to the article and approved the submitted version.

## Conflict of interest

The authors declare that the research was conducted in the absence of any commercial or financial relationships that could be construed as a potential conflict of interest.

## Publisher’s note

All claims expressed in this article are solely those of the authors and do not necessarily represent those of their affiliated organizations, or those of the publisher, the editors and the reviewers. Any product that may be evaluated in this article, or claim that may be made by its manufacturer, is not guaranteed or endorsed by the publisher.
